# Overlooked habitat of a vulnerable gorgonian revealed in the Mediterranean and Eastern Atlantic by ecological niche modelling

**DOI:** 10.1038/srep36460

**Published:** 2016-11-14

**Authors:** Joana Boavida, Jorge Assis, Inga Silva, Ester A. Serrão

**Affiliations:** 1 CCMAR, Centro de Ciências do Mar, Universidade do Algarve, Campus de Gambelas, 8005-139 Faro, Portugal

## Abstract

Factors shaping the distribution of mesophotic octocorals (30–200 m depth) remain poorly understood, potentially leaving overlooked coral areas, particularly near their bathymetric and geographic distributional limits. Yet, detailed knowledge about habitat requirements is crucial for conservation of sensitive gorgonians. Here we use Ecological Niche Modelling (ENM) relating thirteen environmental predictors and a highly comprehensive presence dataset, enhanced by SCUBA diving surveys, to investigate the suitable habitat of an important structuring species, *Paramuricea clavata*, throughout its distribution (Mediterranean and adjacent Atlantic). Models showed that temperature (11.5–25.5 °C) and slope are the most important predictors carving the niche of *P. clavata*. Prediction throughout the full distribution (TSS 0.9) included known locations of *P. clavata* alongside with previously unknown or unreported sites along the coast of Portugal and Africa, including seamounts. These predictions increase the understanding of the potential distribution for the northern Mediterranean and indicate suitable hard bottom areas down to >150 m depth. Poorly sampled habitats with predicted presence along Algeria, Alboran Sea and adjacent Atlantic coasts encourage further investigation. We propose that surveys of target areas from the predicted distribution map, together with local expert knowledge, may lead to discoveries of new *P. clavata* sites and identify priority conservation areas.

Understanding where and how species are distributed is one of the topics of greatest interest in ecology. This is particularly important for structuring species that enhance biodiversity by providing shelter and food sources for other organisms. Having recognized the crucial role of marine biodiversity in ecosystem functioning and services, worldwide conventions and marine policies are being implemented based on scientific data to identify and protect sites of conservation priority (e.g., Convention on Biological Diversity; European Union’s Natura 2000 Network). Among these are extensive areas of long-lived biological structures built by habitat-forming species, including temperate and cold-water octocorals. Yet, there is still little understanding about the factors that control their occurrence at broad distributional scales. This is amplified for species that occur down to depths difficult to study, potentially leaving large areas of distinct benthic communities undiscovered^1^,^2^. Consequently, the relevant spatial data that could be used by marine managers for conservation of vulnerable habitat and species are often incomplete, coarse or inexistent.

Several coral species compose the Mediterranean benthic coralligenous assemblages but few extend into the neighbouring Atlantic Ocean. These biogenic constructions are often dominated by the structuring species *Paramuricea clavata* (Risso, 1826). It occurs in dim light environments of the mesophotic zone (30–200 m depth) and is considered important for fisheries and carbon cycling^3^,^4^. Major threats include mechanical disturbances (e.g., fishing with bottom tending gear, recreational SCUBA diving and anchoring), pollution and increasing seawater temperatures, which have caused mass mortality events throughout the northwestern Mediterranean^5^,^6^,^7^. The species is essentially found in the western Mediterranean Sea, but also in the eastern basin in the Adriatic and Aegean Seas, where it presumably occurs deeper^8^ (V. Gerovasileiou and M. Sini pers. comm.), and in the neighbouring Atlantic Ocean^9^. Although its precise Atlantic distribution is unknown, four records on the coast of Portugal suggest that *P. clavata* may have a wider Atlantic distribution than generally considered^9^,^10^,^11^,^12^. Also, up to 95% of all Mediterranean coralligenous habitat, where *P. clavata* occurs, might still be undocumented in the currently available spatial datasets^1^, particularly in deeper areas further from shore. The actual distributional range of this species, and the factors that determine its range limits remain poorly understood. Ecological Niche Models (ENM) combine observations of species (or habitats) occurrence or density with relevant environmental variables to predict their spatial distributions^13^. They have been used to produce habitat suitability maps and to identify key habitats^14^,^15^. The distribution of a species like *P. clavata* may depend on a combination of biotic and abiotic factors, but the predicted areas of suitable habitat will likely be dependent on the availability, resolution and extent of the data used for modelling^16^. Despite inherent uncertainties, when data are insufficient these tools may serve as a valuable first step in the planning of field surveys, helping to identify the location and overlap of vulnerable habitats, marine protected areas (MPAs) and areas in need of further sampling.

Studies about the ecology of *P. clavata* have focused on regional ecological aspects of the species, mainly centred in the northwestern Mediterranean (refs 17 and 18; but see ref. 19) whereas only limited data exists for a wide area of this species’ distribution (eastern and southern Mediterranean and Atlantic^8^,^9^). The significant lack of continuous information over vast areas has caused effective marine spatial planning measures to be spatially disarticulated and reach limited outcomes^20^. Here we gathered all available occurrences of *P. clavata* across the Mediterranean and adjacent Atlantic coasts. We reviewed scientific and grey literature, online databases, contacted local experts where information was insufficient (eastern and southern Mediterranean basins, Atlantic Morocco), and conducted underwater surveys using SCUBA (in Portugal). Occurrence data and thirteen environmental variables were used to predict the suitable habitat for *P. clavata*, with Boosted Regression Trees, along its full distribution and to determine if predicted physiological limits are coincident with those reported in the literature. This includes spatially representing for the first time an estimate of the suitable habitat for this vulnerable octocoral, from the eastern Mediterranean to the eastern Atlantic coasts, and providing the species physiological limits estimated by the model.

## Results

The environmental predictors produced for Ecological Niche Modelling correlated well with quality-controlled data, reflecting most of the patterns observed in the bottled data (Supplementary Table S1). Temperatures and salinity showed higher accuracy scores (Pearson correlation >0.83) while nutrients showed more moderate values (Pearson correlation ranging between 0.45 and 0.73).

In total 303 occurrence records were collated, of which 23 were from the Atlantic. Eighteen new records were obtained from our underwater surveys (15–90 m depth) and the remaining found in the literature, in online databases and from local experts. Depth varied between 5 and 140 m for the 198 records that reported depth (Supplementary Fig. S2 and Supplementary Dataset S1) and correlated well with EMODNET bathymetry data (correlation 0.77 and mean difference 12.0 ± 7.25 m), whereas other datasets (GEBCO) with coarser resolution were less correlated (0.54, mean difference 17.40 ± 28.1 m). The spatial correlogram for environmental predictors indicates that occurrence records were positively autocorrelated up to 12.5 km (Supplementary Fig. S1). This led to 103 records being kept for modelling from the initial 303, six of which from our diving surveys (Supplementary Dataset S1).

**Figure 1:**
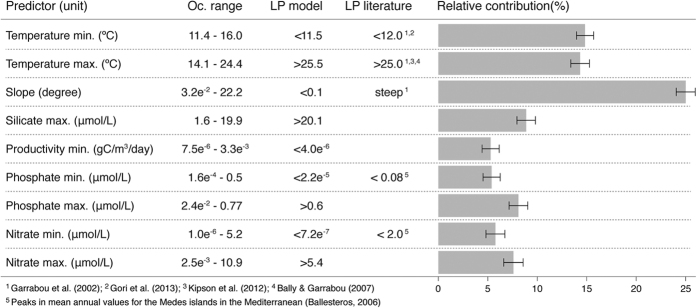
Mean relative contribution (%) of the environmental predictors on model accuracy (TSS), their range of variance in the occurrence records (Oc. range) and limiting-points (LP) captured from the models and from the literature.

ENM showed that the full distribution of *Paramuricea clavata* is best explained by ocean temperatures and slope (Fig. 1). These predictors presented a high relative contribution to the models, while maximum silicate, minimum productivity, phosphate and nitrate had a low contribution (<10%; Fig. 1).

**Figure 2:**
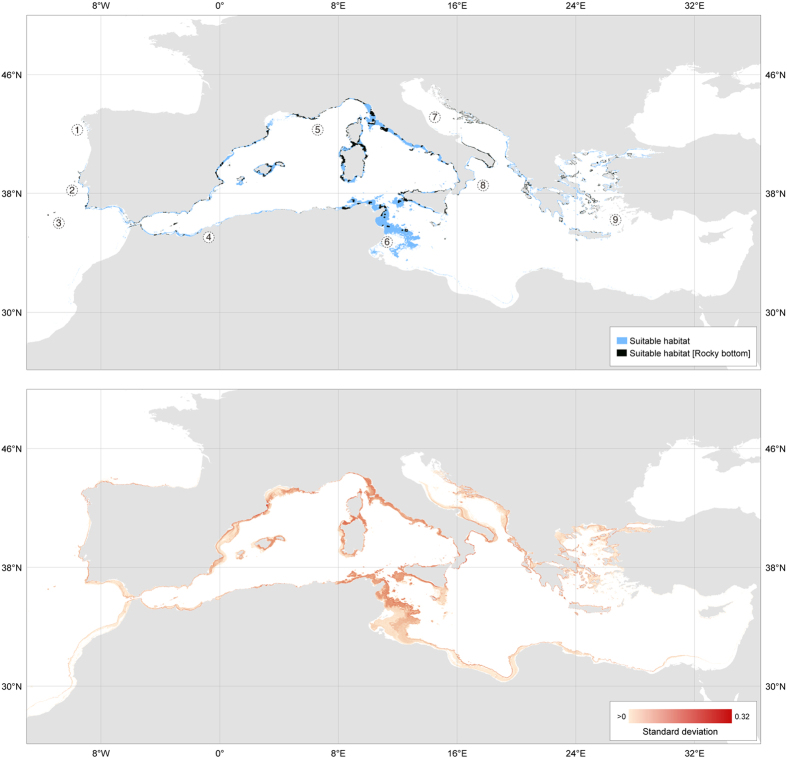
Predicted suitable habitat of *Paramuricea clavata*. Top - The reclassified ensemble of potential habitat of *P. clavata* considering the environmental conditions only (blue) and its interception with rocky bottoms (black); Bottom - Prediction uncertainty (standard deviation) from ensemble modelling. Numbers refer to locations mentioned in the text: 1 - Northwest Iberia (Spain), 2 - West Portugal, including Berlengas islands, 3 - Gorringe Bank (seamounts), 4 - Alboran Sea, including islands and seamounts, 5 - Ligurian Sea, 6 - Gulf of Gabes, Tunisia, 7 -Adriatic Sea, 8 - Ionian Sea, 9 - Aegean Sea. Both images are available for download as GeoTIFF files in link provided in Supplementary Fig. S11. Maps were created with QGIS (www.qgis.org).

The models with higher potential for transferability performed well (mean TSS in cross validation: 0.85 ± 0.01) and explained most of the variance found in the occurrence data (mean deviance explained in cross validation: 0.71 ± 0.02). These allowed producing an ensemble with high predictive accuracy (TSS 0.90; sensitivity 0.96 and specificity 0.94) and low uncertainty (Fig. 2 lower panel).

The predicted suitable habitat, after including the distribution of hard bottom (Supplementary Fig. S6), which is needed for gorgonian settlement, was consistent with known occurrence areas of *P. clavata* but identified areas for which there is no occurrence information and regions down to >150 m depth (e.g., Portugal, around the Strait of Gibraltar, west and eastern basins, mainly in Spain, Morocco, France, Italy, Tunisia and Greece, but also Malta and Turkey). The model identified the majority of probable habitat accompanying the distribution of hard substrate throughout the coastlines of the northwest Mediterranean. In the eastern basin and in the neighbouring Atlantic Ocean (from Morocco to northwest Spain), the predicted distribution is more restricted (Fig. 2 upper panel and Supplementary Fig. S11). Other significant regions of suitable habitat for *P. clavata* are found beyond already known locations, especially in offshore seamounts and islands (e.g., Talbot Bank in the Strait of Sicily, Alboran Island), along sections of Algeria, Tunisia extending to Sicily and along a narrow coastal band at the Ionian Sea (on the Italian side of the Strait of Otranto and in Greek islands). Major discontinuities were found in the southeastern Mediterranean and in the northwestern Adriatic Sea. Additionally, the model predicted the Atlantic putative distributional limits for *P. clavata* located from northwestern Iberia (Galicia) to north of Essaouira on the west coast of Morocco, although with no records there (Supplementary Fig. S2) and few predicted cells. In this southern range caution is needed when interpreting the prediction since there is no information available regarding the distribution of hard substrate (Supplementary Fig. S6). The westernmost habitat areas were predicted at the seamounts of the Gorringe Bank (Portugal) but highly suitable rocky habitat also occurs in western Portugal along the Berlengas islands and the southwest coast, coincident with our field surveys (Fig. 2 upper panel, available for download as GeoTIFF file in Supplementary Fig. S11; Supplementary Fig. S2).

The total estimated suitable habitat area for *P. clavata* is 138 984.6 km^2^ of which 3.9% overlaps with MPAs. When considering rocky bottoms, the estimated suitable habitat is 36 410.3 km^2^ and 14.9% corresponds to MPAs. Outside of the Mediterranean, 8.99% of Atlantic suitable rocky habitat presents some protection level and this includes a very large area over the Gorringe seamount (ca. 23 km^2^), recently classified under the Natura 2000 Network and part of the Marine Strategy Framework Directive (MSFD). Although only 15% of *P. clavata* observations used for modelling were from the Atlantic and the southern Mediterranean, there are considerable areas of predicted suitable habitat there that to date were not surveyed. A large fraction (64%) of the predicted gorgonian hard bottom habitat occurs at mesophotic depths (>50 m) of which only 3.7% is under some protection level. The full distribution on Fig. 2 does not allow discriminating well locally important areas, but the high resolution of the models allow zooming in to see details (Supplementary Fig. S11). For example, our analysis suggests that about 600 km of the Algerian and Tunisian coasts present suitable hard bottom despite sparse records (Supplementary Fig. S2), and also that over 300 km of hard bottom along Portugal, beyond our SCUBA survey sites and literature records, are also highly suitable despite no surveys being available for those areas.

The species predicted response shows a range of optimal conditions for adequate habitat mostly concordant with values obtained from the literature (summarized in Fig. 1). Together, these values delimit the species-specific limiting points (also known as tipping points^21^), here defined as the points at which the build-up of small changes in the environmental variable will culminate on a significant effect for the species (partial dependence and bean plots can be found on Supplementary Figs S8 and S9). In particular *P. clavata* tolerates a wide range of a few environmental variables (e.g., slope, nutrients and productivity) but requires ocean temperatures between 11.5 and 25.5 °C. Specifically for slope, no maximum was detected, indicating that the species might occur in substrates with slope steeper than 22°, the maximum slope value in the presence model cells. The species can grow in vertical walls and overhangs but also in near-horizontal substrates. In general, suitable habitat is unlikely in regions where average minimum monthly temperatures are below 11.5 °C (northern Adriatic and north of Iberia) and maximum temperature is above 25.5 °C (northern Adriatic, Levantine Sea and Gulf of Gabes in Tunisia).

## Discussion

Several studies have highlighted that there are important areas of overlooked marine ecosystems in less studied geographic regions and depths difficult to access^1^,^2^,^22^,^23^,^24^. Our results provide 1) the most comprehensive and updated distributional data of a major ecosystem engineer, *Paramuricea clavata*, extending the geographic range known from previous studies, particularly in the Atlantic (23 occurrences in the Atlantic), 2) the first habitat suitability map for the whole species range, predicting regions of previously overlooked habitat and 3) environmental limiting points for this species obtained from a predictive model that presented high accuracy scores. Adding substrate information contributed to a more realistic prediction, restricting suitable habitat to hard bottom areas, where planula larvae are more likely to settle. This was an original approach since, to our knowledge, it was the first time that substrate type was included in an ecological niche prediction for the full distribution of a marine species (but see ref. 25).

New and previously unknown spatial information about the distribution of *P. clavata* was revealed for the Atlantic region north of the Strait of Gibraltar. The species has been recently described to occur on submarine mountains in the Mediterranean Sea where it was accurately predicted by ENM^26^. A previously unnoted area of potential habitat confined to rocky patches was predicted in the mesophotic zone (>50 m), which comprises most of the modelled gorgonian hard bottom habitat. This includes upwelling regions such as the Alboran Sea and seamounts such as Talbot Bank in the Strait of Sicily. Despite the recent increase of distributional records in the Mediterranean, particularly for corals (e.g., refs 27 and 28), comprehensive spatial analysis of habitat conditions estimating the full distributional range of structural species are mostly missing, more so in neighbouring regions of the Atlantic Ocean (but see ref. 29 for a seagrass). Besides mapping suitable Mediterranean habitat for the gorgonian *P. clavata*, we also report here hotspots where it can be found at high densities in particular Atlantic sites (>1 colony m^−2^, Boavida *et al*.^9^; Supplementary Fig. S10), dominating gorgonian gardens down to >100 m depth in southern and western Portugal (Berlengas islands, in isolated mesophotic reefs off Cape Espichel and Sines and in submerged marine caves - observations from our SCUBA diving surveys, and in the Gorringe seamounts^30^; Supplementary dataset S1). Considering its high biodiversity value, knowing its spatial coverage should be made a priority to help reach conservation goals (for example to establish coherent MPA networks) and tackle the many threats marine habitats face.

*Paramuricea clavata* and other temperate gorgonians are usually associated to areas with a complex bottom relief or steep slopes and areas ranging from high productivity to oligotrophy. The complete environmental range of the species was here modelled by using the full geographical distribution of *P. clavata* with records from the Atlantic. Suitable habitat was found to occur across narrow stretches of coastlines and other relatively shallow but complex topographic ocean features (near shelf breaks and on seamount summits), strongly associated to slope, a surrogate indicative of hard substrate^31^. The species requires a specific range of ocean temperatures (11.5 to 25.5 °C), which remarkably matches previous experimentation work conducted in the Mediterranean^17^,^32^. Areas where maximum temperatures exceed the tolerated values for prolonged periods were predicted to be largely unsuitable for *P. clavata*, such as the Levantine Sea where the species has been confirmed absent^33^. Nutrients contributed more moderately to the model (<10%) with different weights, but because there are high correlations between minimum and maximum values (>90%; Supplementary Fig. S7), it is difficult to determine the individual role of each predictor on the ecological niche of the species. Other studies predicted coral and gorgonian habitat suitability to be primarily related to oceanographic and terrain variables, including ocean temperatures, slope and also nutrients (e.g., refs 22, 34, 35, 36). Sites with adequate slope and temperature combined with flow velocity and moderate to high productivity are important for maintaining effective prey capture and the metabolic rates of corals^4^,^32^,^37^. Topography and bathymetry influence food availability for benthic suspension feeders by interacting with water flow: there is an enhanced water transport of food particles, which happens either by local (tidal) advection coupled with water re-suspension at the reef tops or by convective (vertical) currents from deep nutrient-rich waters^38^.

BRT has been used recently to explain and predict with high accuracy species distributions and past or future range alterations in the marine environment (e.g., refs 39 and 40), since this technique presents great flexibility to handle distinct data types and provides accurate explanation and prediction results^41^. Yet, there is an obvious dependence of models from the spatial distribution of the record points used. Leveraging the environmental drivers that most strongly shape a species’ distribution is not a trivial task and requires sound knowledge about its biology and habitat interactions. The environmental variables used in our study do not represent an exhaustive set of predictors likely to influence the suitable habitat for *P. clavata* and other relevant factors may be used (e.g., other terrain derived variables, biotic variables^16^). Whilst predictors had a general good agreement with the quality-controlled dataset (Supplementary Table S1), nutrients were less correlated with bottled data. This is likely due to the higher temporal variability of these predictors (e.g., upwelling regions^42^) and to the forced relationship between a daily observation (bottled data) and mean monthly extremes (predictor data). The application of high-resolution data at global scales is a transversal limitation for marine modellers. As spatially complete datasets with higher resolution become available, models will achieve a higher explanatory and predictive capacity. While spatially and depth biased data result in imperfect predictions, standardized basin-wide surveys for our entire study area, including at depth, are not realistically tenable. Here, some areas of suitable hard bottom habitat were predicted beyond presence observations (extrapolation). Habitat areas that were predicted for the largely under-surveyed African coast (Atlantic and Mediterranean), mesophotic areas and seamounts should be interpreted with caution due to the limited number of observations there, and to the lack of hard bottom data for the Atlantic coast of Morocco (Supplementary Dataset S1, Figs S2 and S6). Extrapolated areas are found in narrow stretches of coastline along west Algeria and a few rocky patches in the Gulf of Gabes (south Tunisia). *P. clavata* was further predicted at the Strait of Otranto (southern Adriatic Sea), on the east Ionian (Greece) and south Tyrrhenian Seas but currently the only surveys conducted there were too deep to register this upper mesophotic species^28^. In the Atlantic, where the thermal niche falls within the Mediterranean thermal range (Supplementary Fig. S9), the model predicted within this area and not beyond it (23 record points along >700 km of coastline, including the offshore Gorringe Seamounts). Still, areas off northwest Iberia (Portugal and Spain) were predicted to present suitable habitat for the species in regions where it is unknown to occur, but where the sister species *P. grayi* is present. The region presents considerable areas of high-energy circalittoral rock (www.meshatlantic.eu) and water temperature within its tolerated range^43^,^44^. Yet, the predominant longshore drift from North to South, with inversions only during the non-reproductive season (winter^45^), may prevent it from colonizing otherwise suitable habitat. In contrast to predicted regions where it might be absent, all regions where the species is known to occur are well predicted by the model. This highlights the need for field surveys where the ensemble presents higher standard deviations and where field information is missing. Yet, despite uncertainties in identifying the climatic envelope of the species, it includes southern distribution limits supported by the prediction of areas that have suffered mass die-offs^17^. Estimating areas based on model outputs carries some uncertainty, yet, taken with caution, this is a tool within our reach to inform management while other relevant and higher resolution data remain elusive. Within these limitations, results presented are informative and can be used for decision-making.

It remains a considerable challenge to map this vulnerable habitat. Data for the Atlantic, in particular along the Moroccan coast, is very scarce or completely unavailable, and still much remains to be known about the distribution of invertebrate biodiversity along the African coasts of the Mediterranean and in deep sites. Here we predicted a putative rocky habitat area of >25 000 km^2^ (including the seabed down to >150 m and the neighbouring Atlantic). Although a considerable proportion of gorgonian hard bottom habitat is protected under a MPA status (ca. 15%), relevant areas are still unrecognized by any legal conservation status, supporting that there is insufficient overlap between MPA and areas of concern for biodiversity. We highlight the under protection of over 90% of the predicted mesophotic hard bottom habitats (>50 m depth) and areas of the studied Atlantic coasts (Portugal and Morocco). There, nearly 9% of predicted rocky habitat is under some protection, however, a large fraction of this figure is the classification of sites in the framework of Natura 2000, at a national level, which currently either do not include any management measures (Gorringe Seamount, classified in 2015) or that target marine birds but not the benthic habitats where *P. clavata* occurs (Berlengas archipelago). Cumulative impacts on species caused by direct anthropogenic stressors such as breakage by fishing gear and sea surface temperature changes may severely affect populations locally but can also have a global impact at the species capacity to adapt, such as if unique genetic traits of range edge populations (e.g., Atlantic) and from deeper sites become lost for the global species pool. Conservation planning benefits from considering species global ranges rather than just local scales. Our results can also be used in future surveys to conduct more cost-effective samplings, especially in areas that represent a greater challenge due to its inaccessible location (e.g., offshore seamounts, mesophotic reefs) or where data is insufficient (e.g., north African coasts). A problem in large-scale habitat management is often the lack of comparable data or no data at all across large-scale boundaries. By comprising the entire distribution of a major structural species, our results can be incorporated in conservation management approaches at scales beyond political and geographical borders.

## Material and Methods

### Ethics Statement

All field surveys did not involve disruptive sampling and thus no specific permits were required.

### Study location

The study area included the entire Mediterranean Sea and the adjacent Atlantic Ocean, from 24**°**71N and 36**°**E to 51**°**85N and 18**°**W, corresponding to the known distributional range of the red gorgonian, *Paramuricea clavata* (Supplementary Dataset S1 and references therein). This area spans about 4 000 km horizontally and encompasses the entire littoral coastlines, islands, underwater features such as submarine mountains and continental shelf slopes. Populations of *P. clavata* are found growing over rocky substrates from about 15 m down to about 200 m depth, but may occur shallower under low light conditions^46^ (http://natura2000.eea.europa.eu/Natura2000/SDF.aspx?site=ESZZ16002; Supplementary Dataset S1).

### Environmental data

Multiple environmental predictors were produced for ecological niche modelling based on the known ecology and physiological tolerances of *P. clavata*. These were produced with three-dimensional profiles describing monthly averages from 2000 to 2010 of ocean temperature, salinity, nutrients (phosphate, nitrate and silicate), net primary productivity and current velocity, obtained from the Global Ocean Physics Reanalysis and the Biogeochemistry Non Assimilative Hindcast Simulation (ORAP and PISCES; www.marine.copernicus.eu; Supplementary Table S1). We used the highest resolution global bathymetry dataset available (0.002°; European Marine Observation Data Network; Supplementary Table S1). The environmental dataset profiles were gridded to match the resolution of bathymetry using trilinear interpolation (e.g., ref. 47) weighting the information of location (longitude and latitude) and depth of each cell of bathymetry, within the vertical coverage between 15 and 200 m depth. Long-term monthly extremes were averaged and used as environmental predictors (e.g., the long-term average of the warmest month; Supplementary Table S1). Slope was computed with bathymetry using the “terrain” function in R package “raster” (R Foundation for Statistical Computing, 2014), which safely differentiates between coordinate systems.

Environmental predictors were compared with quality-controlled water data (e.g., ref. 42) obtained from the World Ocean Atlas and the Global Ocean Data Analysis Project^48^,^49^,^50^,^51^ (www.nodc.noaa.gov; http://cdiac.ornl.gov/oceans/glodap; Supplementary Table S1). To this end, environmental data were produced as described previously, but using the location and depth of each water bottle sample (Quality-control data). We adopted this approach since few locations of water samples matched the cells of our study region. The paired relationships between interpolation product and bottle data were determined with Pearson’s correlation. Quality-control data on net primary productivity and currents were not available for comparison.

### Distribution data

We compiled a database with distributional records (presence data from 1975 to 2015) of *P. clavata* from the literature (Supplementary Dataset S1), online species databases (GBIF; OBIS; MARBIS; Natura 2000 Network) and by contacting local experts whenever insufficient data was available in the literature (Supplementary Dataset S1 and references therein). Depths reported in the collated data were compared with depths inferred from the bathymetry grid. Records at improbable depths (e.g., deeper than 200 m) or falling on land (e.g., refs 42 and 52) were removed. The database was completed by field surveys because recent studies^9^,^10^,^11^ and divers’ observations (http://www.deepreefs.com) indicated that the species could be present in the Atlantic Ocean, along the south and west coasts of Portugal. To pinpoint the distribution along the Portuguese coastline, we performed SCUBA and rebreather dives from south to west Portugal (15–90 m depth at Tavira, Portimão, Lagos, Sagres, Sines, Cape Espichel and Berlengas islands; Fig. 2), noting its presence. Dive survey coordinates can be found in Supplementary Dataset S1. North of Berlengas the species is absent and from the Galicia region northwards a sister-species is present, *P. grayi*^53^. All records of occurrence were gridded to match the spatial resolution of environmental data (0.002°; Supplementary Fig. S2).

To reduce the effects of spatial autocorrelation we tested the relatedness of environmental predictors within the occurrence records, as a function of distance using a Mantel test in R with package “ecospat” (9999 permutations). When more than one record occurred at distances shorter than the minimum significant autocorrelated distance, only one record was kept.

### Cross-validation and Ecological Niche Modelling

We used a cross-validation implementation using a non-random partition of distinct spatial groups (described below^54^) to identify a set of parsimonious models from several possible models that presented a high potential for accurate prediction of the distribution of *P. clavata* within its ecological domain (i.e., spatial transferability). Over-fitting was reduced by discarding predictors that have null contributions in cross-validation and using specific model parameterization (see below). We adopted Boosted Regression Trees (BRT), an ensemble technique that combines the strength of fitting multiple regression trees with a boosting algorithm, in a forward, stage-wise fashion (for a detailed explanation see ref. 55). Because BRT uses binary occurrence data (presence and absence) but accurate absence data were unavailable for *P. clavata*, we generated pseudo-absences through a Mahalanobis Distance function^56^. The function uses the presences and normalized environmental predictors^57^ to determine an Environmental Suitability Map (ESM; Supplementary Fig. S3), specifying for each cell how distant (in probability of occurrence) their local habitat is from the niche optimum. Pseudo-absences were chosen from the ESM cells that presented a probability ≤0.2. This low threshold favours sensitivity (correctly predicted presences), over specificity (correctly predicted absences), a desirable criterion in conservation to avoid rare/important species being wrongly classified^58^. Lower thresholds, e.g., 0.1^59^, may lead to over-prediction of the niche by selecting pseudo-absences from a very narrow set of conditions in those map cells. Pseudo-absences were then structured as environmentally dissimilar cells using K-means clustering on the normalized predictors; the k clustering parameter used was the number of presence records (see ref. 60 for details). To reduce estimation biases introduced by a putative unbalanced distribution of occurrence records and to restrict the modelling exercise to the spatial extent of the species distribution, pseudo-absences were randomly selected following the underlying weight of the sampling effort (i.e., target-group information^61^). Weighting used a kernel density surface considering the spatial distribution of occurrence records throughout the study region (e.g., ref. 62; Supplementary Fig. S4).

Model transferability was assessed with a 10-fold cross-validation by partitioning the distribution data (records of occurrence and pseudo-absences) into 10 non-random longitudinal bands. In this process models were iteratively fitted with all combinations of non-correlated predictors (Pearson’s correlation <|0.8|; e.g., ref. 63) while withholding one spatial band at each iteration to test predictive performances on the withheld band (as largely recommended^54^). The withheld band is used as a surrogate for an independent dataset (e.g., under-sampled region).

The predictive performance of the BRT model was optimized by the 10-fold cross-validation in the training dataset with four parameters: bag fraction, corresponding to the fraction of the training data selected for the next fitted tree; learning rate (*lr*), responsible for shrinking the contribution of each tree added to the model; number of trees (*nt*), which corresponds to the number of iterations occurred in each model; and tree complexity (*tc*), which regulates the number of nodes in a tree and, hence, the level of interaction between predictors^41^,^55^. To determine the best combination of values for all model parameters, we examined the change in deviance over *lr* values of 0.01, 0.005, 0.001 and 0.0005, *tc* from 1 to the number of predictors in each model, and a bag fraction of 0.5^55^, ensuring that at least 1000 trees were fitted per model. Deviance reduction was used as a measure of model performance. To further reduce potential over-fitting, minimum predictors were forced to produce positive monotonic responses in the models, whereas maximum predictors were forced to negative effects. The predictive performance of each model was verified with True Skill Statistics (TSS^64^) by comparing the predicted distributions with the withheld test data, while applying a threshold that maximizes the ability to detect true presences and absences, calculated as the sum of sensitivity and specificity, respectively.

The mean decrease in deviance obtained when adding each predictor to all alternative models was used to determine predictor contribution. Independent non-parametric Kruskal-Wallis rank tests were used to identify the combinations of predictors with highest potential for transferability (null hypothesis of no differences in mean TSS; alpha = 0.05). The final map was produced by ensemble modelling, a procedure that uses a median function to merge the resulting predictive surfaces of all models identified as equally accurate during cross-validation (with the best combination of predictors). In this step, the full dataset of occurrences was used. A reclassification was applied to the final map to ensure maximum sensitivity and specificity, so that it reflects predicted presences and absences and not a probability of occurrence. The accuracy of the final prediction was assessed with TSS, sensitivity and specificity, and its uncertainty was computed by producing a surface of the standard deviation of the ensemble. The reclassified ensemble was intercepted with available information about the distribution of rocky bottoms^65^ throughout the species distribution (EMODnet portal www.emodnet-seabedhabitats.eu; Supplementary Fig. S6).

Bean plots were produced by intersecting the occurrence records with the environmental predictors that best described the ecological niche of *P. clavata* (relative contribution to the models >5%). Limiting points were inferred by extracting the extreme values (minimum and maximum depending on the predictor) from the reclassified ensemble predictive surface (i.e., which set the extreme conditions for the occurrence of the species; e.g., ref. 63). Partial dependence plots (Suplementary Fig. S8) were also produced to show the effect of each variable on the response after accounting for the average effect of all other predictors, by following the implementation of ref. 55.

The total predicted habitat area of the species was estimated with and without intersecting with the hard substrate surface (Supplementary Fig. S6). Furthermore we compared how the predicted suitable habitat of *P. clavata* overlaps with existent Marine Protected Areas, Natura 2000 and national designated sites, considered here as a unique network and referred to as MPAs (we excluded the large Pelagos Sanctuary, which is directed at Marine Mammals) across the entire geographical range studied (Supplementary Fig. S12). We used a dataset of Mediterranean MPAs made available by the online database MAPAMED (http://www.medpan.org/en/mapamed) and complemented it with information from national reports, legislation and databases for Portugal, Spain and Morocco (http://www.icnf.pt/portal/naturaclas/cart; http://www.redeuroparc.org/descargasmapas.jsp). A total of 162 areas were included, from 19 countries.

Niche modelling and area calculations were performed in R version 3.1.1 (R Foundation for Statistical Computing, 2014) using the packages adehabitat, dismo, doParallel, ecospat, gbm, gstat, parallel, raster, rgdal, SDMTools, sm and sp.

## Additional Information

**How to cite this article**: Boavida, J. *et al*. Overlooked habitat of a vulnerable gorgonian revealed in the Mediterranean and Eastern Atlantic by ecological niche modelling. *Sci. Rep.* **6**, 36460; doi: 10.1038/srep36460 (2016).

**Publisher’s note:** Springer Nature remains neutral with regard to jurisdictional claims in published maps and institutional affiliations.

## Supplementary Material

Supplementary Information
