# Supplementary material for: Overlooked habitat of a vulnerable gorgonian revealed in the Mediterranean and Eastern Atlantic by ecological niche modelling
Source: Sci Rep. 2016 Nov 14;6:36460. doi: 10.1038/srep36460 (PMC5107895; doi:10.1038/srep36460)
Supplement: Supplementary Information [file srep36460-s1.pdf]

Supporting information

Scientific Reports

Overlooked habitat of a vulnerable gorgonian revealed in the Mediterranean and Eastern Atlantic by ecological niche modelling

Joana Boavida, Jorge Assis, Inga Silva, Ester A. Serrão

Contents

Dataset S1. Records of occurrence used to model the ecological niche of *Paramuricea clavata*.
Table S1. Environment predictors used in ecological niche modelling.
Figure S1. Spatial autocorrelation of environmental predictors within occurrence records.
Figure S2. Spatial distribution of the records of occurrence used to model the ecological niche of *Paramuricea clavata*.
Figure S3. Habitat suitability of *Paramuricea clavata* computed with Mahalanobis distance.
Figure S4. Probability surface to select pseudo-absence records.
Figure S5. Habitat suitability of *Paramuricea clavata* predicted with Boosted Regression Trees.
Figure S6. Distribution of benthic rocky reefs, coralligenous habitats and marine caves.
Figure S7. Correlation matrix for the environmental predictors use for niche modelling.
Figure S8. Partial dependence plots.
Figure S9. Bean plots and environmental limiting-points.
Figure S10. Image of dense gorgonian aggregation in Portugal.
Figure S11. GeoTiff maps with the predicted *Paramuricea clavata* distribution and standard deviation (link and DOI for download).
Figure S12. Marine Protected Areas of the Mediterranean Sea (excluding Pelagos Sanctuary) and neighboring Atlantic (Portugal, Spain and Morocco).
References.

Dataset S1. Records of occurrence of *Paramuricea clavata*. Includes site name, georeferenced coordinates, depth, which records were used for modelling and the source of the record point; available for download at Figshare under the follow link:

https://dx.doi.org/10.6084/m9.figshare.3102616.v1

Table S1. Environmental predictors used in Ecological Niche Modelling. Includes predictor units, native resolution (the number of vertical layers refers to the depth range between 15 and 200 m), predictor range within the study region, accuracy (given by the Pearson correlation between predictor and quality-controlled water bottle data from the World Ocean Atlas [Locarnini et al., 2013; Zweng et al., 2013; Garcia et al., 2013; www.nodc.noaa.gov] and the Global Ocean Data Analysis Project [GLODAP; Sabine et al., 2005; http://cdiac.ornl.gov/oceans/glodap]; asterisk for significant correlations under a linear model; alpha=0.001) and source. Minimum and maximum values for each predictor were calculated as the mean monthly extremes (see Methods for details).

Predictor	Unit	Native Resolution	Range	Accuracy	Source
Temperature min.	ºC	0.25º (21 vertical layers)	7.2 - 18.5	0.83 * (n=16870)	ORAP [1]
Temperature max.	ºC	0.25º (21 vertical layers)	11.0 - 29.0	0.88 * (n=16981)	ORAP [1]
Salinity min.	PSS	0.25º (21 vertical layers)	20.1 - 39.1	0.99 * (n=967845)	ORAP [1]
Silicate min.	µmol/L	0.25º (21 vertical layers)	1.1 - 74.4	0.57 * (n=9859)	PISCES [2]
Silicate max.	µmol/L	0.25º (21 vertical layers)	1.6 - 133.3	0.45 * (n=9859)	PISCES [2]
Phosphate min.	µmol/L	0.25º (21 vertical layers)	$2.2e^{-5}$ - 1.0	0.73 * (n=9779)	PISCES [2]
Phosphate max.	µmol/L	0.25º (21 vertical layers)	$9.6e^{-3}$ - 1.1	0.65 * (n=9779)	PISCES [2]
Nitrate min.	µmol/L	0.25º (21 vertical layers)	$1.4e^{-7}$ - 11.8	0.67 * (n=8919)	PISCES [2]
Nitrate max.	µmol/L	0.25º (21 vertical layers)	$3.5e^{-6}$ - 12.5	0.63 * (n=8919)	PISCES [2]
Net Primary Productivity min.	$gC/m^3/day$	0.25º (21 vertical layers)	0 - $1.2e^{-2}$		PISCES [2]
Net Primary Productivity max.	$gC/m^3/day$	0.25º (21 vertical layers)	$8.6e^{-7}$ - 0.1		PISCES [2]
Currents min.	m/s	0.25º (21 vertical layers)	$4.0e^{-5}$ - 0.6		PISCES [2]
Slope	degree	0.002º	0 - 67.7		EMODNET [3]

[1] Global Ocean Physics Reanalysis ECMWF ORAP5.0, Copernicus Marine and Environment Monitoring Service (www.marine.copernicus.eu)
[2] Biogeochemistry non assimilative hindcast simulation PISCES, Copernicus Marine and Environment Monitoring Service (www.marine.copernicus.eu)
[3] European Marine Observation Data Network (EMODnet) Seabed Habitats project (www.emodnet-seabedhabitats.eu)

Figure S1. Spatial autocorrelation (Mantel r value) of environmental predictors within occurrence records as a function of distance. Black circles indicate values significantly different from zero. Dashed line depicts the selected distance (first distance where autocorrelation is not significantly different from zero).

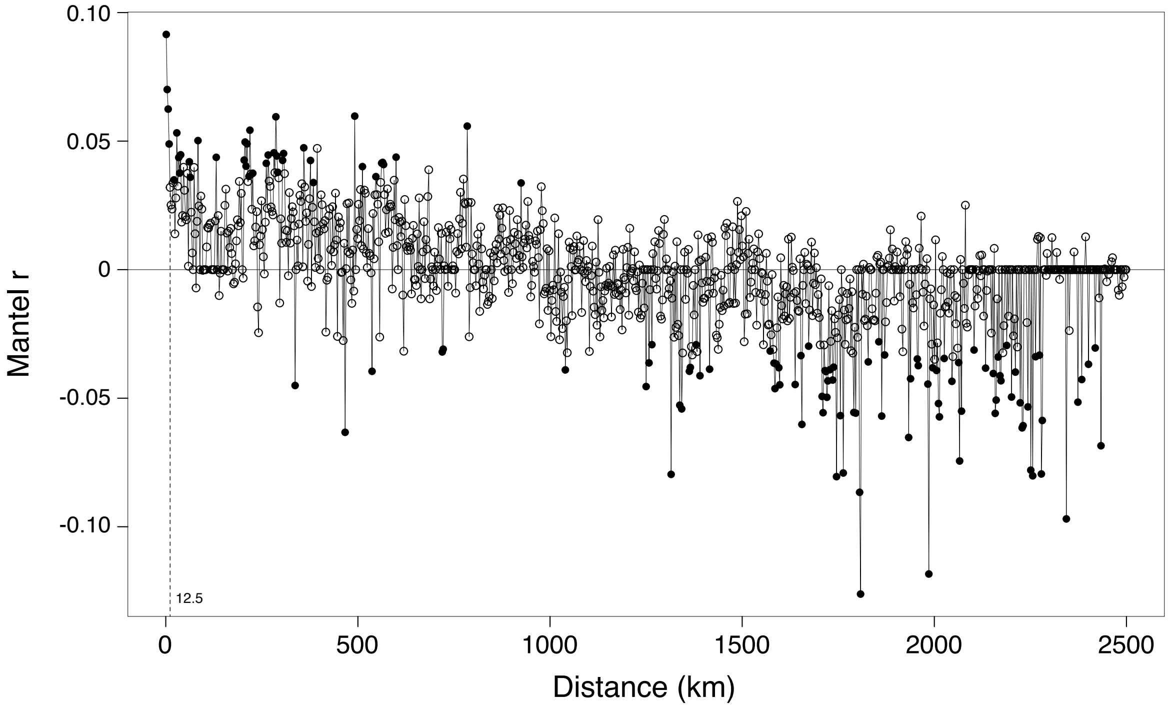

Figure S2. a) Spatial distribution of the records of occurrence used to model the ecological niche of *Paramuricea clavata*. Circles - occurrence records collated from Dataset S1. Dots - occurrence records used for modelling (selected after spatial autocorrelation analysis; see Methods for details). Square depicts region of field surveys (SCUBA and rebreather dives). b) Bottom right inset - Histogram of depth for all occurrence records. Map was created using QGIS version 2.4 (www.qgis.org).

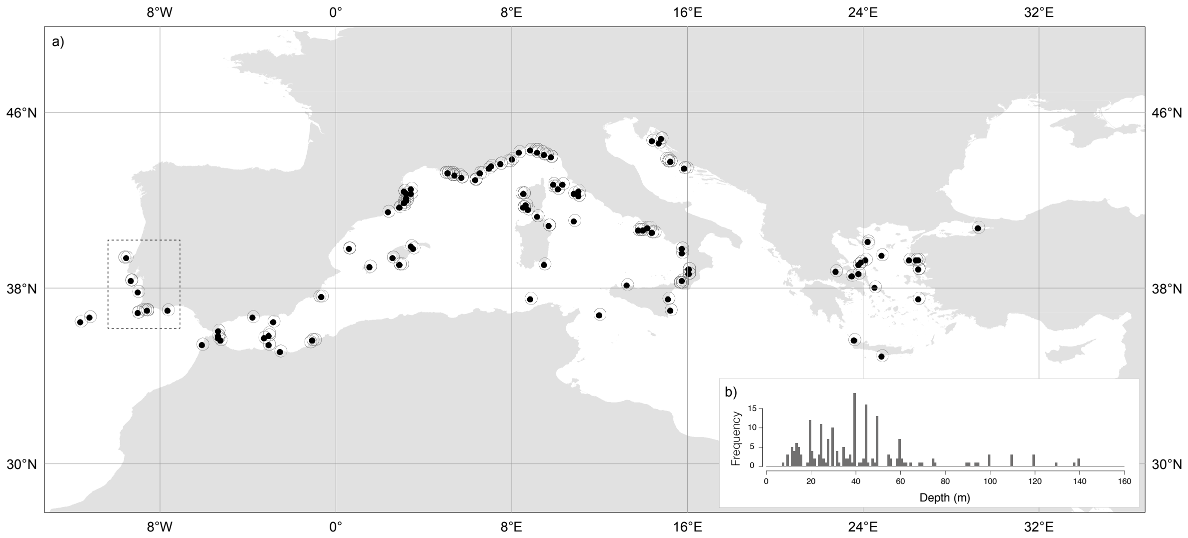

Figure S3. Habitat suitability of *Paramuricea clavata* computed with the Mahalanobis distance function. Used in the selection of pseudo-absences. Map was created using QGIS version 2.4 (www.qgis.org).

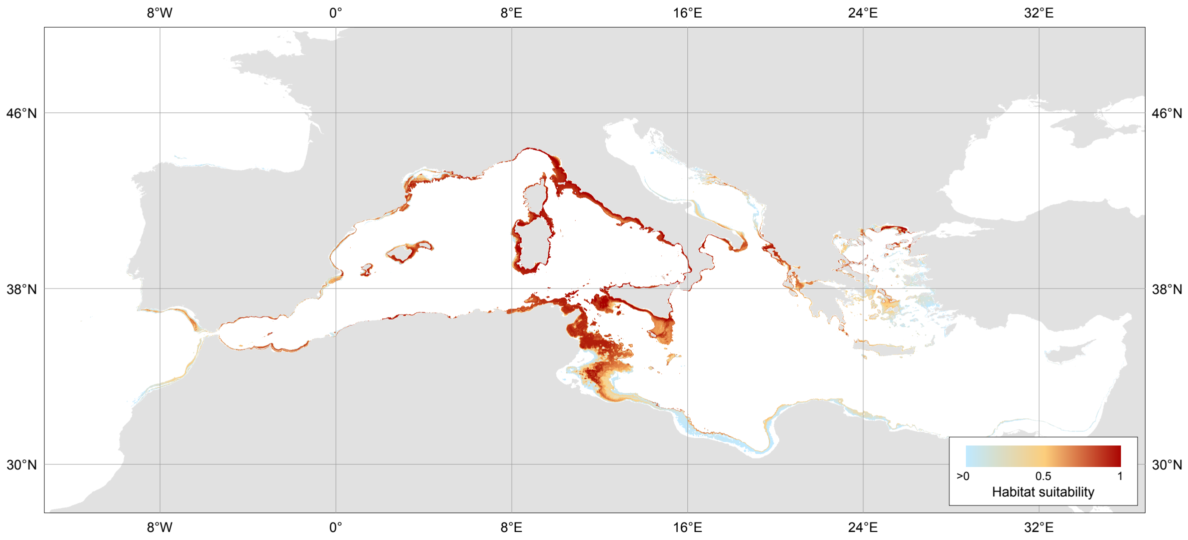

Figure S4. Probability surface (kernel density estimation) used to select pseudo-absence records. This was computed to reduce putative biases introduced by disparities in sampling effort (e.g., presence of more thoroughly sampled and undersampled regions. Map was created using QGIS version 2.4 (www.qgis.org).

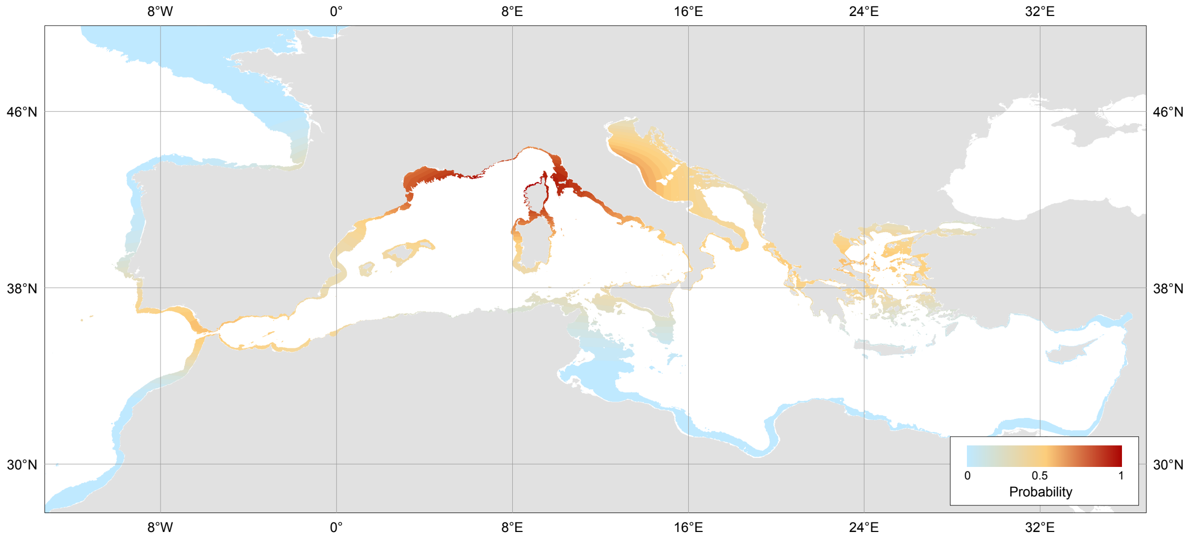

Figure S5. Habitat suitability of *Paramuricea clavata*. Predicted with Boosted Regression Trees by ensembling the best transferrable models identified in the cross validation procedure. Map was created using QGIS version 2.4 (www.qgis.org).

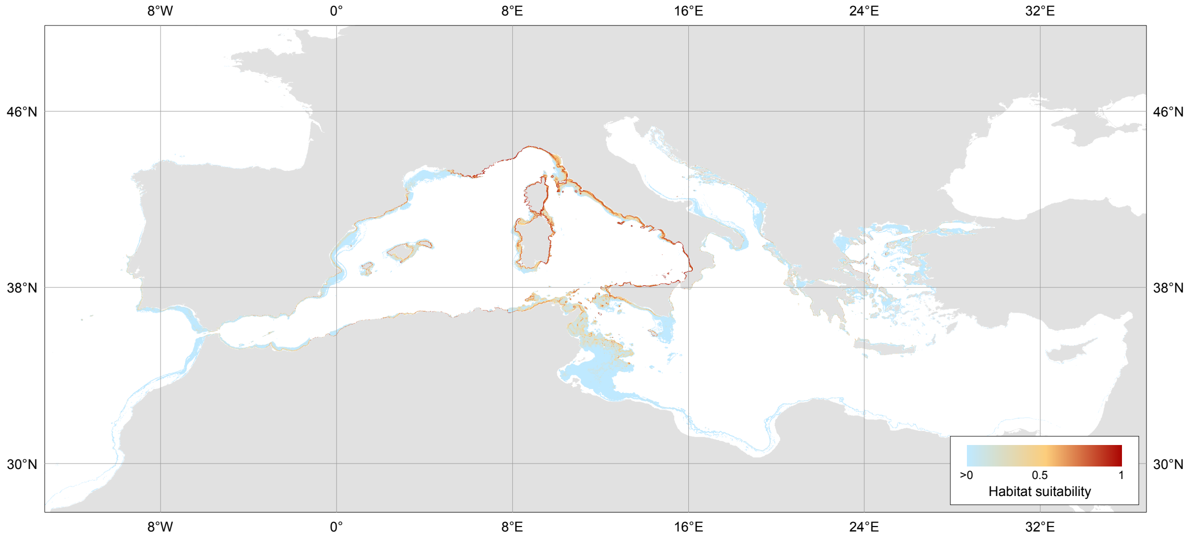

Figure S6. Distribution of benthic rocky reefs, coralligenous habitats and marine caves. Inferred from the Seabed Habitats project (European Marine Observation Data Network, www.emodnet-seabedhabitats.eu) funded by the European Commission's Directorate-General for Maritime Affairs and Fisheries (DG MARE) and from the data available in Giakoumi et al. (2013). Dashed area depicts regions for which there is no information available. Map was created using QGIS version 2.4 (www.qgis.org).

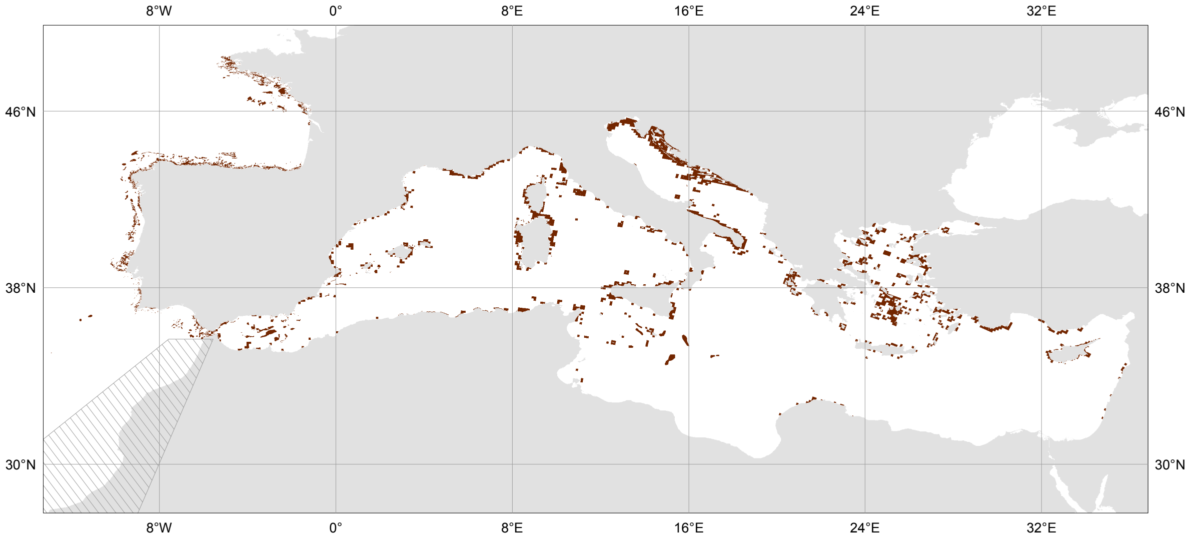

Figure S7. Correlation matrix for the environmental predictors use for niche modelling. The diagonal shows the name and histogram of each predictor. The right side o the plane shows the correlation coefficient (Pearson) between predictors, while the left side plots the correlation between predictors.

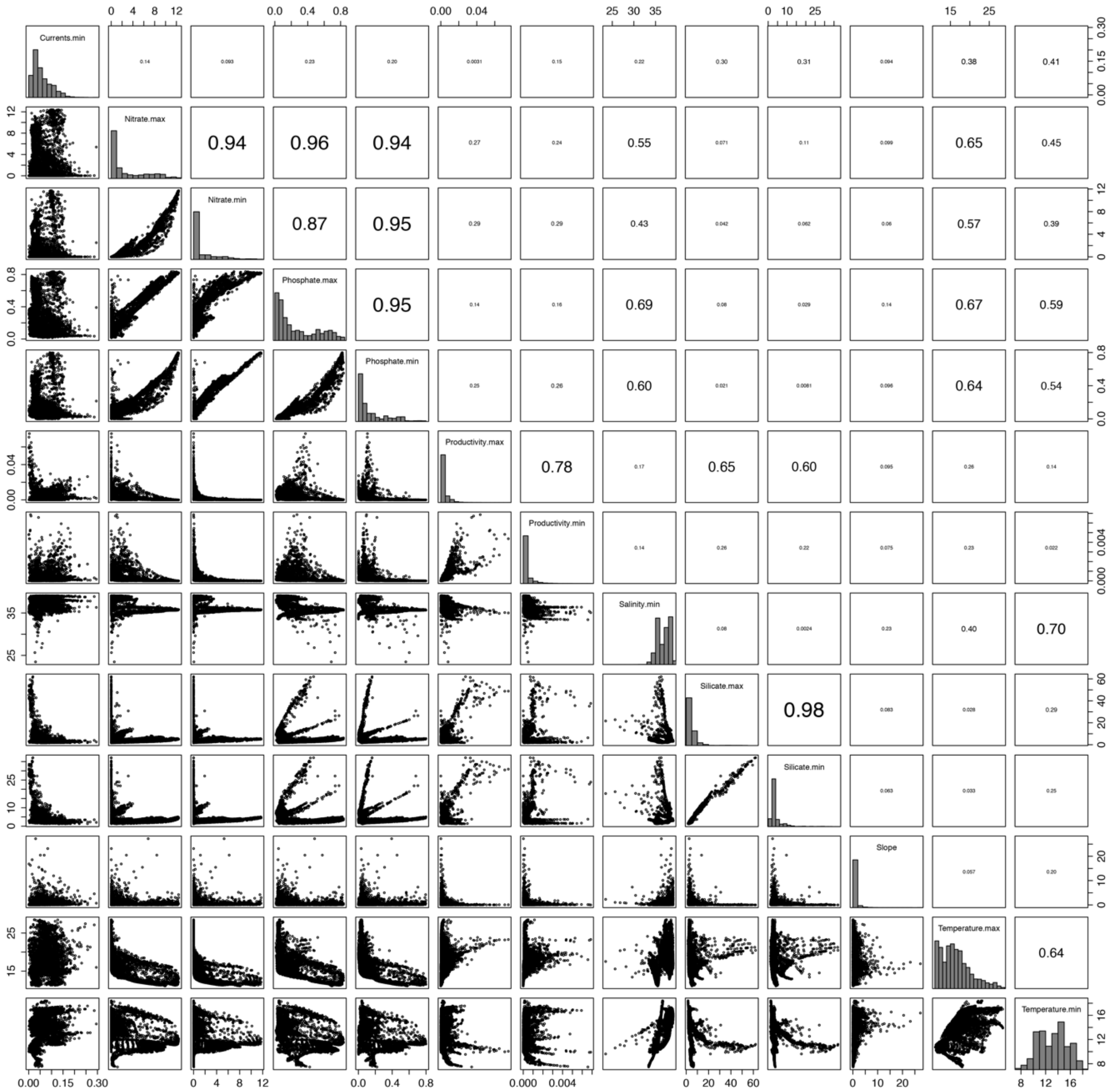

Figure S8. Partial dependence plots for variables with contributions >5%. Shows the relationship between predictor variables and the probability of occurrence of *Paramuricea clavata* as modeled by BRT ensemble modelling. The shape of the fitted functions shows the overall effect of the variable across its full range (x-axis) in the response (probability of occurrence) represented on a relative scale (y-axis). Positive values in the fitted functions (y-axis) have a positive effect on the probability of occurrence of *P. clavata*, given the effect of all other predictors.

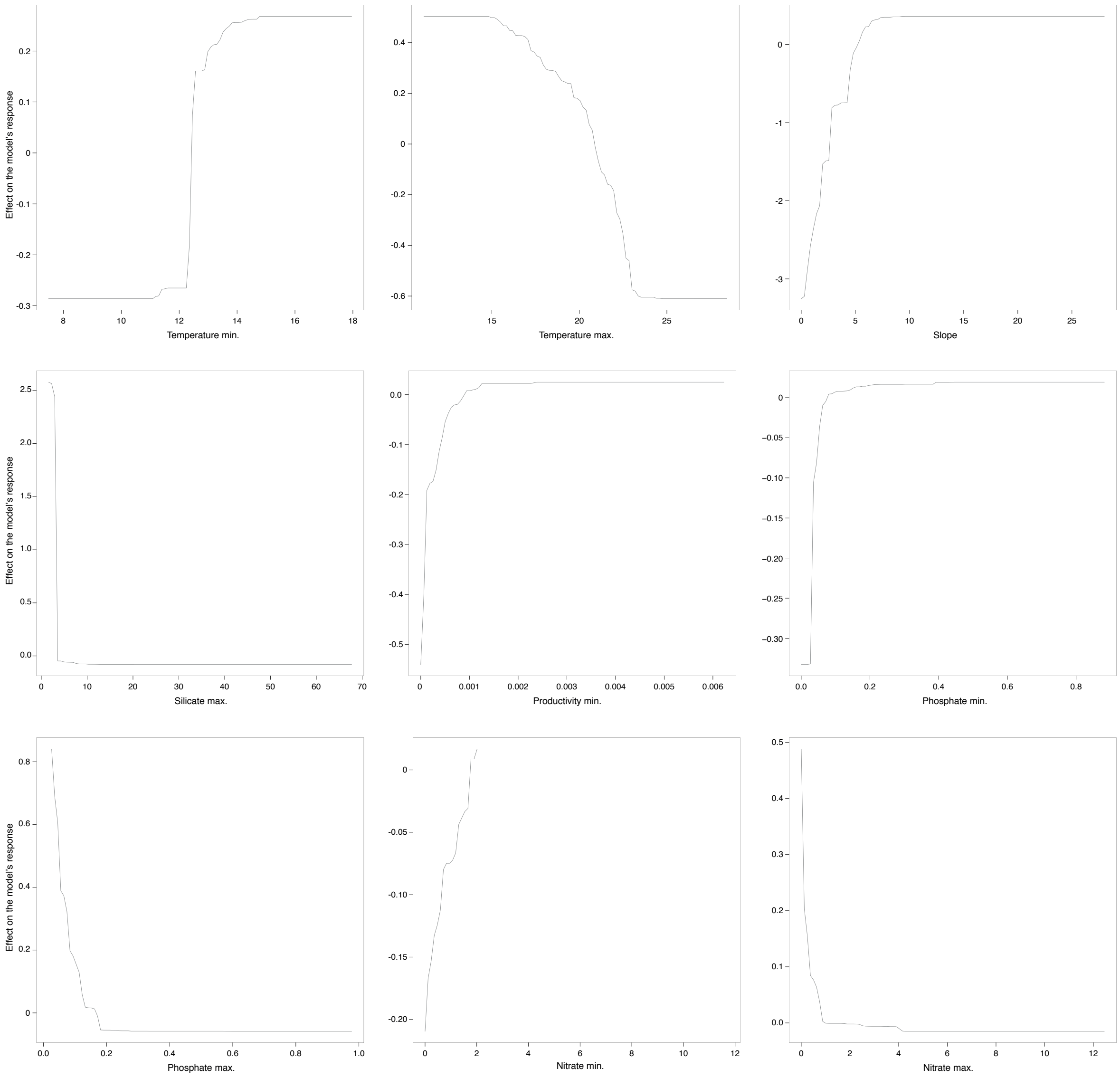

Figure S9. Bean plots of records of occurrence intersected with the environmental predictors with > 5% contribution to the ecological niche model. The horizontal lines in each bean refer to individual occurrence records. Upper right values show the environmental limiting-points (predictor extreme values) captured from the reclassified habitat suitability surface, when using a threshold maximizing sensitivity and specificity (e.g., Assis et al., 2016). Dashed squares show the Atlantic range.

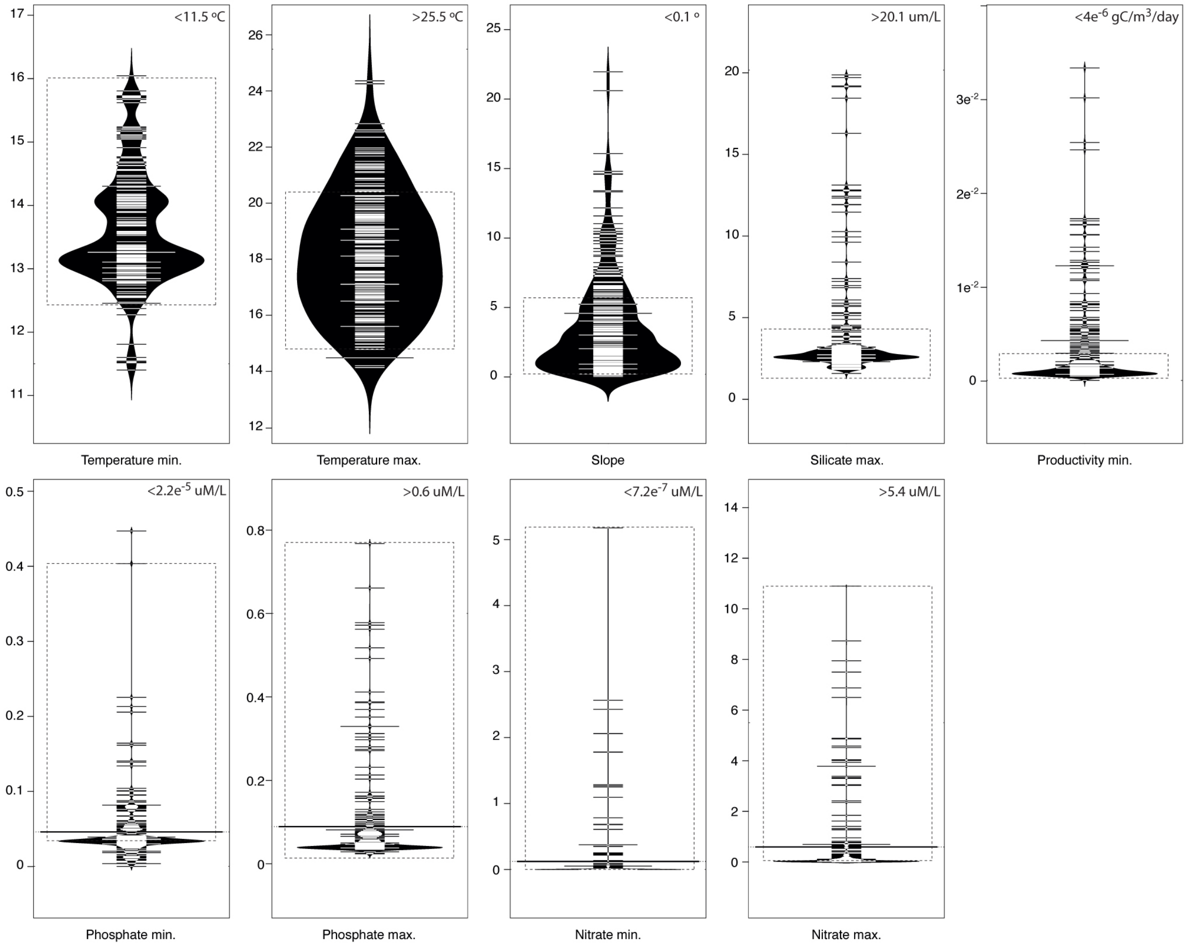

Figure S10. Dense *Paramuricea clavata* aggregation (violet gorgonian) in west Portugal at 50 m depth. The image is a snapshot from a video and presents some distortion due to the wide angle lens. Credits: Delfim Machado (www.deepreefs.com).

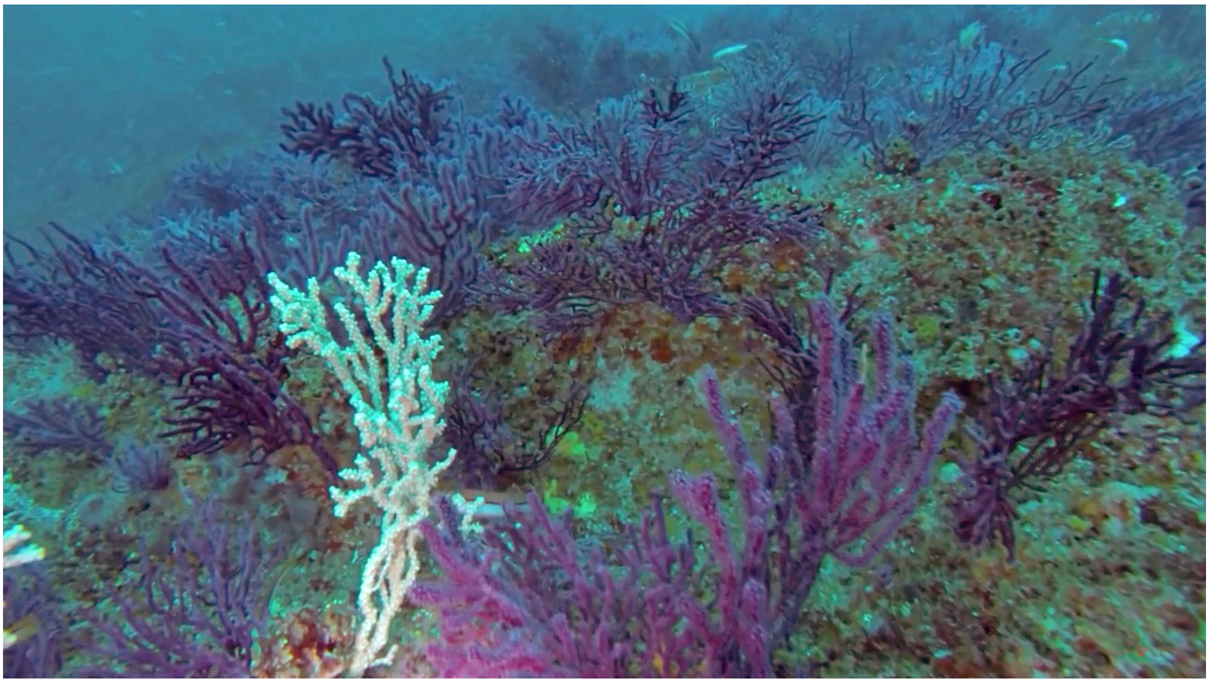

Figure S11. Georeferrenced maps from ensemble BRT modelling available for download at Figshare under the following links: **Habitat suitability:** http://dx.doi.org/10.6084/m9.figshare.3466712; **Standard deviation:** http://dx.doi.org/10.6084/m9.figshare.3470489.

Figure S12 - Marine Protected Areas of the Mediterranean Sea (excluding Pelagos Sanctuary) and neighbouring Atlantic (Portugal, Spain and Morocco) included in our analysis. Very small polygons may not be visible. Map was created using QGIS version 2.4 (www.qgis.org) and MPA areas available from the 2012 dataset of the MAPAMED database (http://www.mapamed.org) developed by MedPAN and RAC/SPA, Europarc-Spain (http://www.redeuroparc.org/descargasmapas.jsp and http://www.redeuroparc.org/observatorio/descargas) and the Portuguese Nature Conservation Institute (http://www.icnf.pt/portal/naturaclas/cart).

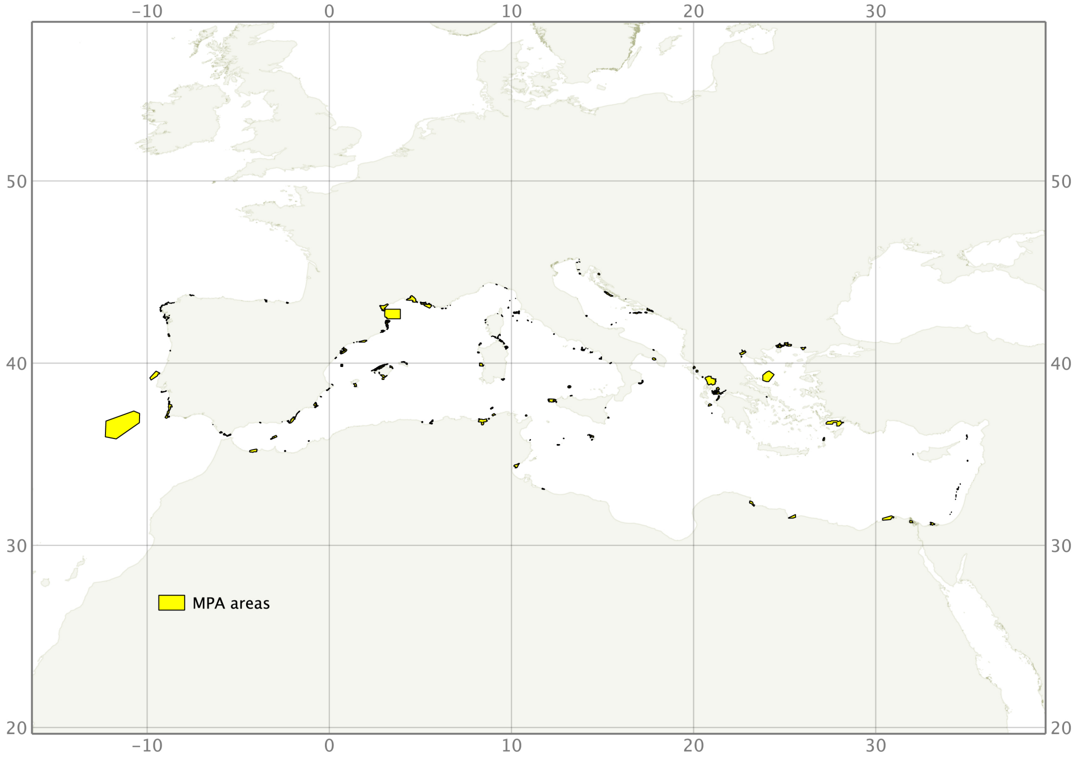

References

Assis, J. *et al.* Deep reefs are climatic refugia for genetic diversity of marine forests. *J. Biogeogr.* **43,** 833–844 (2016).

Garcia, H. E. *et al. WORLD OCEAN ATLAS 2013 Volume 4: Dissolved Inorganic Nutrients (phosphate, nitrate, silicate)*. *NOAA Atlas NESDIS 76* **4,** (NOAA Atlas NESDIS 76, 2013).

Giakoumi, Sylvaine et al. "Ecoregion-Based Conservation Planning in the Mediterranean: Dealing with Large-Scale Heterogeneity." *PLoS ONE* 8.10 (2013).

Locarnini, R. A. *et al. World Ocean Atlas 2013, Volume 1: Temperature*. *NOAA Atlas NESDIS 73* **1,** (NOAA Atlas NESDIS 73, 2013).

Sabine, C. L. *et al. Global Ocean Data Analysis Project: Results and Data*. *ORNL/CDIAC-145, NDP-083* (2005). doi:10.3334/CDIAC/otg.ndp083

Zweng, M. M. *et al. World Ocean Atlas 2013, Volume 2: Salinity*. **2,** (NOAA Atlas NESDIS 74, 2013).
